# The Guanine-Nucleotide Exchange Factor SGEF Plays a Crucial Role in the Formation of Atherosclerosis

**DOI:** 10.1371/journal.pone.0055202

**Published:** 2013-01-25

**Authors:** Thomas Samson, Jaap D. van Buul, Jeffrey Kroon, Christopher Welch, Erik N. Bakker, Hanke L. Matlung, Timo K. van den Berg, Lisa Sharek, Claire Doerschuk, Klaus Hahn, Keith Burridge

**Affiliations:** 1 Department of Cell Biology and Physiology, and Lineberger Comprehensive Cancer Center, University of North Carolina at Chapel Hill, Chapel Hill, North Carolina, United States of America; 2 Department of Molecular Cell Biology, Sanquin Research and Landsteiner Laboratory, Academic Medical Center, University of Amsterdam, Amsterdam, The Netherlands; 3 Department of Pharmacology, University of North Carolina at Chapel Hill, Chapel Hill, North Carolina, United States of America; 4 Department of Biomedical Engineering and Physics, Academic Medical Center, Amsterdam, The Netherlands; 5 Department of Blood Cell Research, Sanquin Research and Landsteiner Laboratory, Academic Medical Center, University of Amsterdam, Amsterdam, The Netherlands; 6 Pulmonary Diseases and Critical Care Medicine, University of North Carolina at Chapel Hill, Chapel Hill, North Carolina, United States of America; 7 McAllister Heart Institute, University of North Carolina at Chapel Hill, Chapel Hill, North Carolina, United States of America; Heart Center Munich, Germany

## Abstract

The passage of leukocytes across the endothelium and into arterial walls is a critical step in the development of atherosclerosis. Previously, we showed *in vitro* that the RhoG guanine nucleotide exchange factor SGEF (Arhgef26) contributes to the formation of ICAM-1-induced endothelial docking structures that facilitate leukocyte transendothelial migration. To further explore the *in vivo* role of this protein during inflammation, we generated SGEF-deficient mice. When crossed with *ApoE* null mice and fed a Western diet, mice lacking SGEF showed a significant decrease in the formation of atherosclerosis in multiple aortic areas. A fluorescent biosensor revealed local activation of RhoG around bead-clustered ICAM-1 in mouse aortic endothelial cells. Notably, this activation was decreased in cells from SGEF-deficient aortas compared to wild type. In addition, scanning electron microscopy of intimal surfaces of SGEF^−/−^ mouse aortas revealed reduced docking structures around beads that were coated with ICAM-1 antibody. Similarly, under conditions of flow, these beads adhered less stably to the luminal surface of carotid arteries from *SGEF* ^−/−^ mice. Taken together, these results show for the first time that a Rho-GEF, namely SGEF, contributes to the formation of atherosclerosis by promoting endothelial docking structures and thereby retention of leukocytes at athero-prone sites of inflammation experiencing high shear flow. SGEF may therefore provide a novel therapeutic target for inhibiting the development of atherosclerosis.

## Introduction

Atherosclerosis is characterized by the successive influx of pro-inflammatory monocytes and lymphocytes into the arterial wall resulting in localized inflammation, accumulation of lipids, fibrosis and cell death. These regions of local thickening are known as atherosclerotic plaques [1]. ICAM-1 is a prominent endothelial cell surface receptor for leukocyte recruitment and studies in mice have shown that interfering with the function of this receptor reduces the formation of atherosclerotic lesions [2]–[4]. Previously, we found that ICAM-1 clustering on endothelial cells relocates the Rho guanine nucleotide exchange factor (GEF) SGEF (Arhgef-26) to the plasma membrane where it leads to activation of the Rho family GTPase RhoG [5]. Subsequently, RhoG activation contributes to the formation of actin rich protrusions on the dorsal surface of endothelial cells [5], known as transmigratory cups or docking structures [6]; [7]. These cups are thought to serve as intermediate docking structures that provide mechanical support for adhered leukocytes before transmigration (reviewed in [8]; [9]). However, it is not known if these structures are involved in pathology such as atherosclerosis. Also, whether Rho-GEFs in general are involved in this process is unclear. Using SGEF-deficient and wildtype mice, we show here that SGEF contributes to the progression of atherosclerosis. Examining the interaction of beads coated with antibodies against ICAM-1, we find that although initial adhesion of the beads to the walls of isolated arteries is identical in the SGEF null mice, exposure to flow dislodges more beads from SGEF null than from wildtype arteries. Consistent with these results, the SGEF null vessels and endothelial cells reveal lower RhoG activation and decreased docking structure formation than their wildtype counterparts. Our results suggest that the reduced formation of docking structure in mice lacking SGEF contributes to the decreased susceptibility of these mice to develop atherosclerosis.

## Results

**Figure 1:**
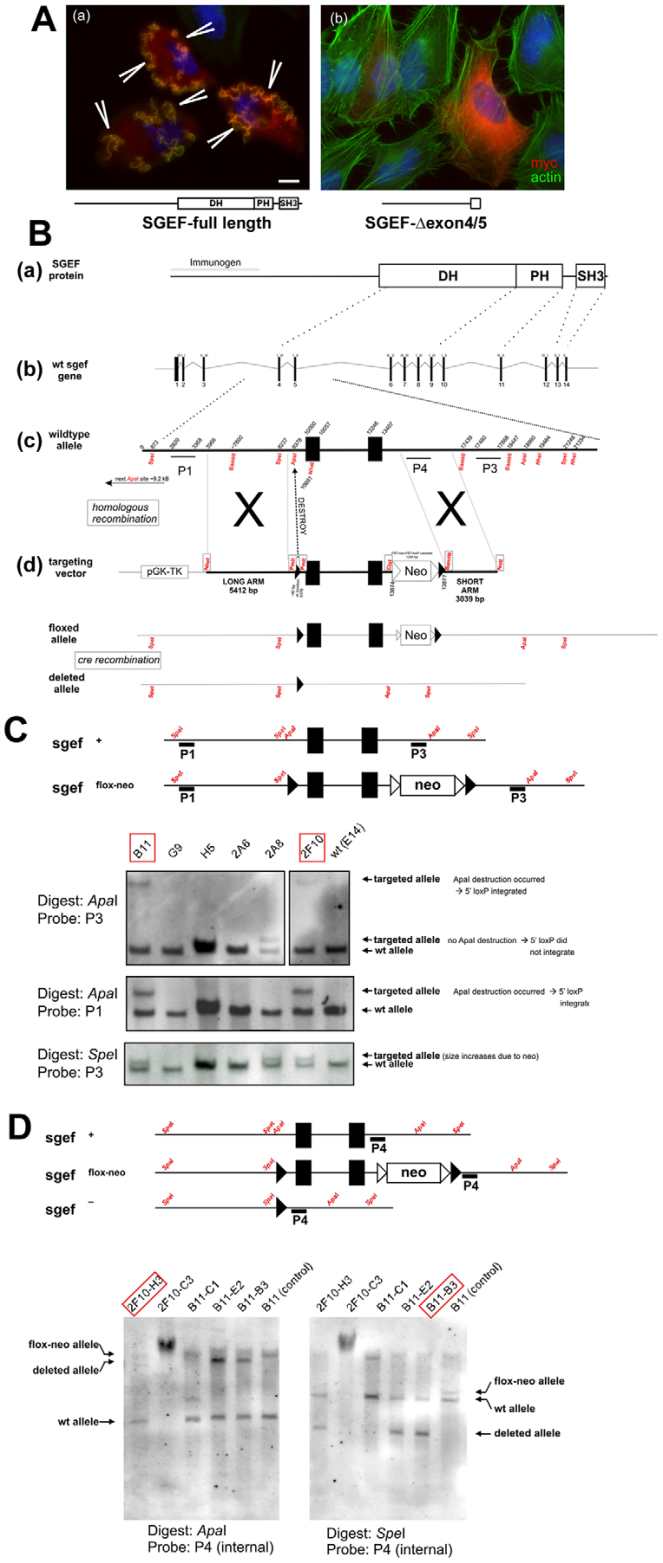
Generation of SGEF-deficient mice. **A:** HeLa cells were transfected with myc-tagged full-length SGEF (a) or mutated SGEF (Δexon4/5) (b). SGEF was stained with an anti-myc Ab (red) and F-actin was visualized in green using Phalloidin. In the left panel, three positive cells in red are shown; in the right panel, one positive cell in red is shown. Bar: 10 µm. **B:** Schematic presentation of the murine SGEF protein (**a**) and gene (**b**). Domain structure of SGEF protein and genomic structure of the SGEF gene are shown according to the database. Latin numbers indicate exon numbers. Roman numbers indicate the last and first codon position of each exon, respectively. (c) *Enlarged area of exons 4/5.* Numbers represent base pairs counted from 10 kB upstream of exon 4. The positions of cutting sites for selected DNA-endonucleases are indicated. The locations of the external probes P1 and P3 and the internal probe P4 are indicated as bars. (**d**) Schematic representation of the targeting vector. Filled triangles represent loxP sites; open triangles represent frt sites. Framed DNA-endonuclease recognition sites were introduced into the genomic DNA by the targeting construct. An endogenous *Apa*I site (at 9378) was destroyed by homologous recombination with the targeting construct and served to detect homologous integration of the construct. **C:** *Identification of successfully targeted ES-cell clones.* After transfecting ES-cells with the targeting construct and neomycin/ganciclovir selection, Southern blotting was performed with DNA from selected ES cell clones using the indicated endonucleases. The binding sites of the probes used (P1 and P3) were located externally on both sides of the genomic area covered by the targeting construct. The diagram at the top shows the location of the endonuclease recognition sites and the probes in the wild type allele and the recombined allele, respectively. ES cell clones B11 and 2F10 were selected and used for subsequent steps. **D:** *Identification of successful deletion of exons 4/5*. Southern blotting was performed with DNA from ES cell clones isolated after cre-transfection. The used probe P4 was located internally within the genomic area covered by the short arm of the targeting construct. The diagram at the top shows the location of the endonuclease recognition-sites and the probe P4 in wild type, targeted and cre-deleted alleles.

**Figure 2:**
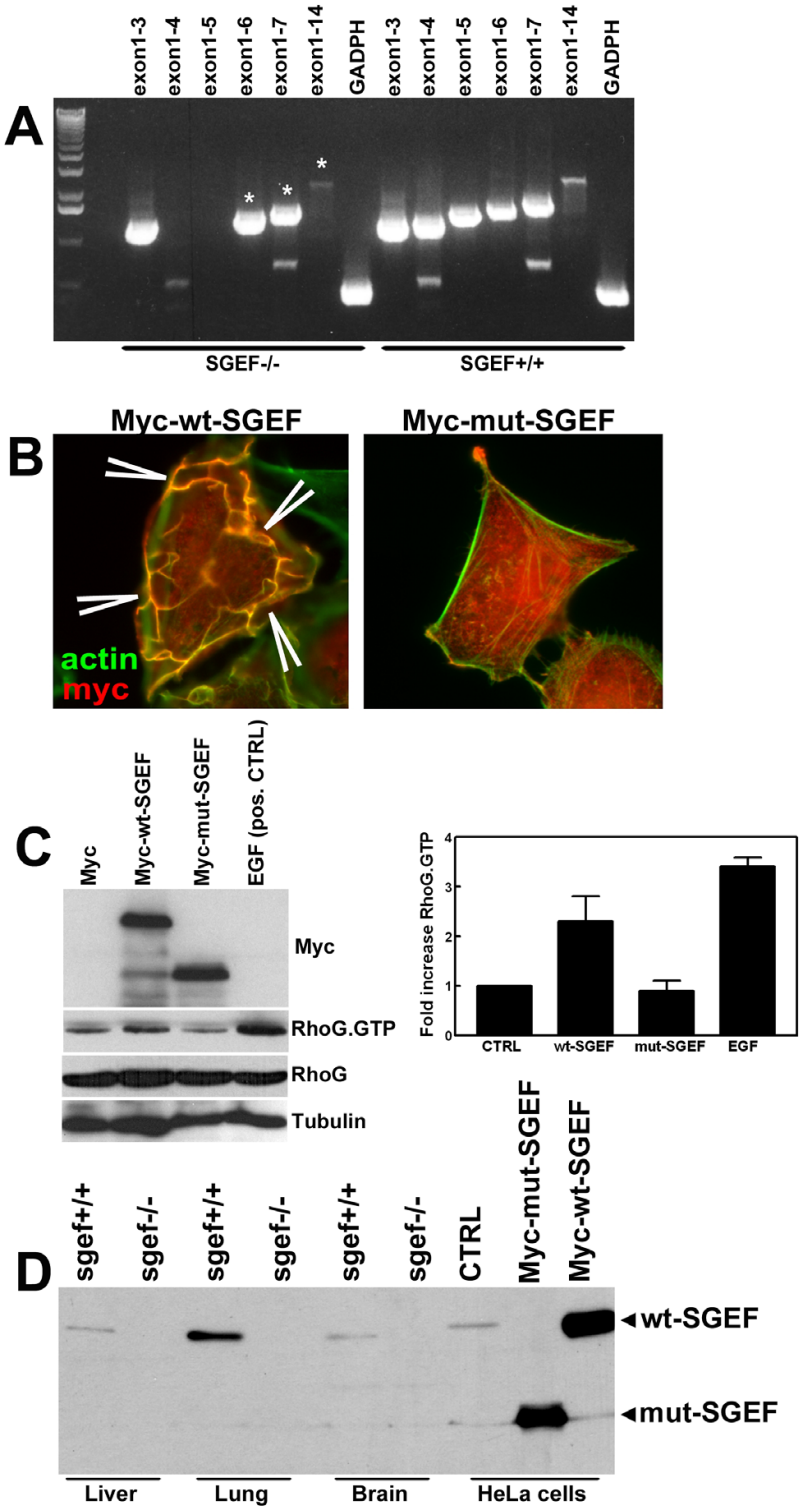
Basic phenotyping of SGEF deficient mice. **A:** RT-PCR from mouse liver mRNA using primers which bind in exon 1 and in different downstream exons of mouse SGEF as indicated. RT-PCR bands from SGEF^−/−^ animals marked with an asterisk are ∼200 bp smaller compared to wild type. **B and C:** SGEF ex1-14 cDNA cloned from RT-PCRs from SGEF^+/+^ and SGEF^−/−^ mice was over expressed in HeLa cells. The transfected HeLa cells were analyzed for dorsal ruffle formation (arrowheads) (**B**) and the activity levels of RhoG (**C**). Graph at the right shows fold increase of Rho.GTP loading upon transfection or treatment as indicated. This experiment was repeated 4 times. **D:** Expression of SGEF in different mouse tissues. The polyclonal antibody used was raised against a 200 amino acid stretch at the N-terminus of murine SGEF.

Since silencing RhoG or SGEF respectively were shown to reduce leukocyte transmigration in a tissue culture model [5], we hypothesized that deficiency of SGEF in mice would also affect inflammatory responses by interfering with the proper formation of apical cup structures. To test this hypothesis, we generated a mouse line deficient in SGEF. *In vitro* over-expression experiments using a mutated SGEF expression construct indicated that deletion of a cDNA stretch encoded by exons 4/5 of the murine SGEF gene, resulted in a non-functional protein (Figure 1A). Based on this, we generated mice that lack exons 4 and 5 of the mouse SGEF gene (Figure 1B–D). Intercrossing of heterozygote animals (SGEF^+/−^) resulted in all three possible genotypes, indicating that SGEF deficiency did not have lethal consequences during embryogenesis. Animals of all genotypes and gender are viable and fertile and do not show differences in leukocyte counts (Table S1) or other obvious phenotypes (followed up to 18 months of age). Genotype ratios of offspring after crossing SGEF^+/−^ animals matched the expected Mendelian distribution and did not suggest an embryonic lethal phenotype (Figure S1). RT-PCR analysis showed that mutated SGEF mRNA isolated from SGEF^−/−^ mice was still detectable (Figure 2A). However, sequencing and expression of the SGEF cDNA cloned from SGEF^−/−^ mice showed that the introduced mutation led to a frame shift proximal to the DH domain, resulting in the expression of a protein fragment that lacked GEF activity. This was confirmed by immunofluorescence experiments that showed that SGEF-Δ4/5 did not induce typical dorsal ruffles, whereas the full length construct did, as was also shown previously by our group [10] (Figure 2B). Also biochemical studies showed that SGEF-Δ4/5 was unable to activate endogenous RhoG, whereas full length SGEF did (Figure 2C). Furthermore, when using a polyclonal anti-SGEF antibody directed to the N-terminus that recognizes the mutated form of SGEF expressed exogenously by HeLa cells, we did not detect any SGEF protein fragment in Western blots of organ lysates from SGEF^−/−^ mice (Figure 2D). These data show that mutated SGEF mRNA is subject either to nonsense mRNA-mediated decay or that any expressed truncated SGEF protein is highly unstable. Taken together, deletion of exons 4/5 of the SGEF gene resulted in animals that are completely devoid of any functional SGEF protein.

**Figure 3:**
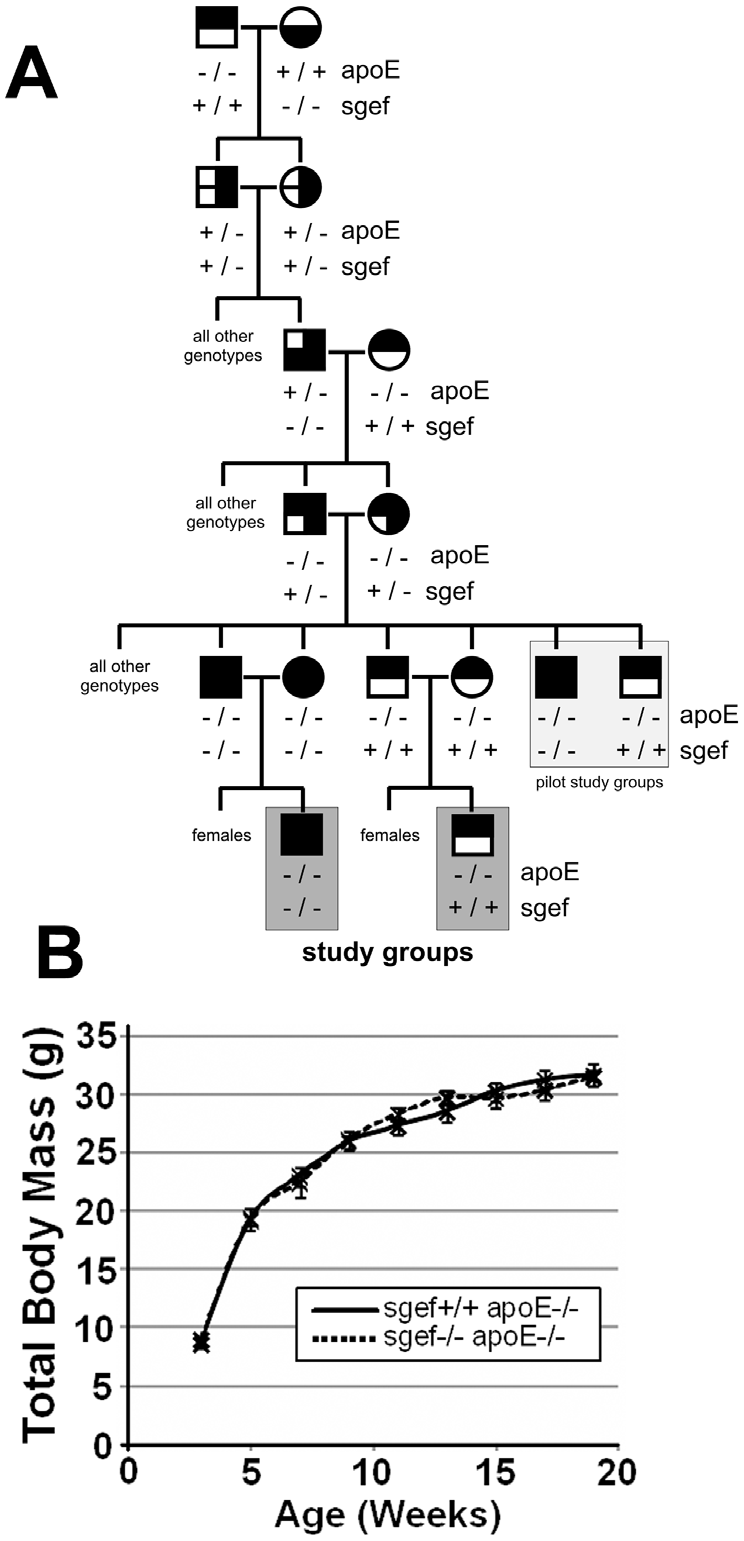
Generating an SGEF-deficient and ApoE-deficient mouse model to examine development of atherosclerosis. A: Breeding scheme to generate SGEF/ApoE double knock-out mice. Litter mates from breeding SGEF^+/−^ApoE^−/−^ mice were used in a pilot study (marked with light grey). Animal groups highlighted in dark gray were used for all subsequent studies to characterize formation of atherosclerosis. **B:** Total body mass of study animals at different ages. Error bars represent SEM.

**Figure 4:**
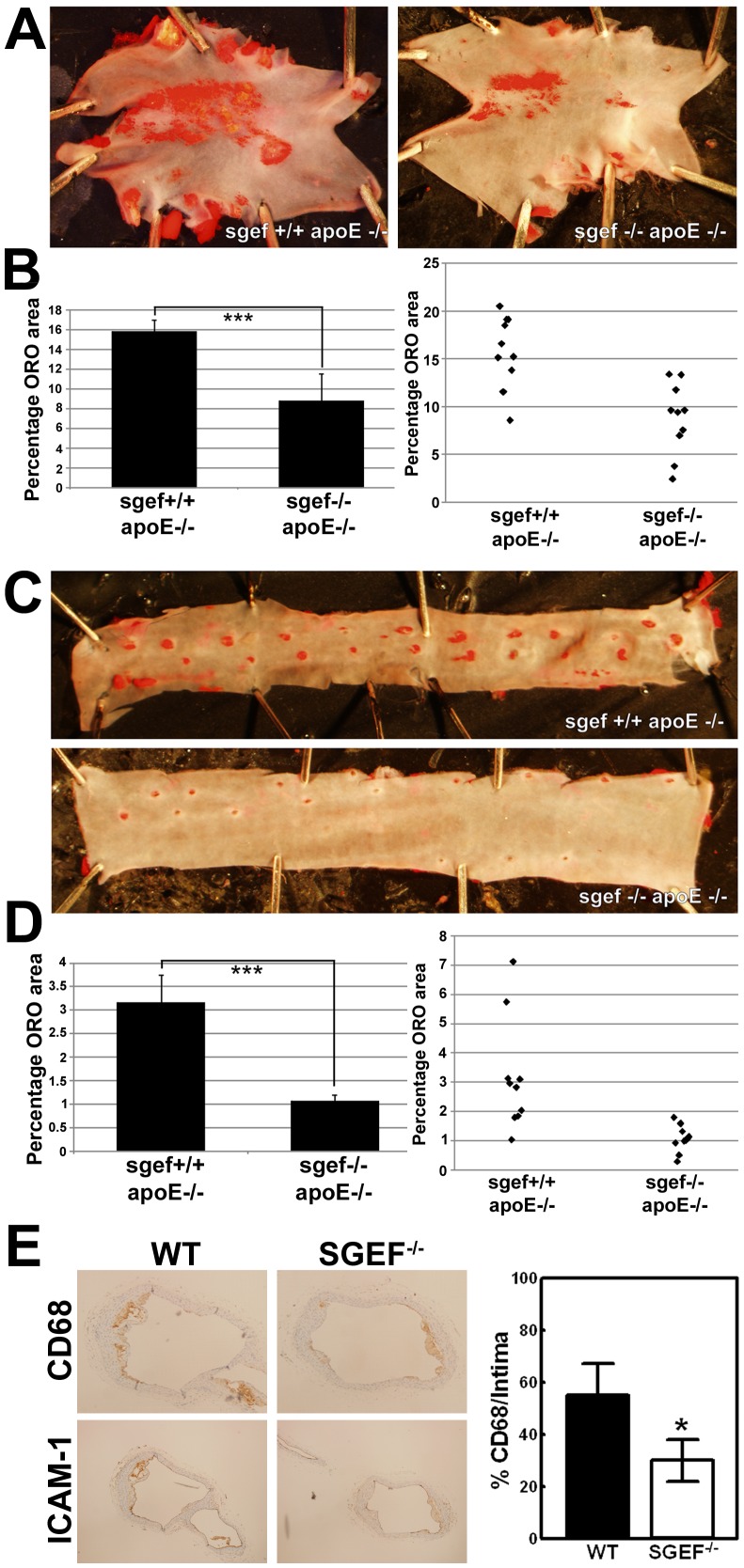
Reduced atherosclerotic plaque formation in SGEF deficient mice fed a Western diet. SGEF^+/+^ApoE^−/−^ and SGEF^−/−^ApoE^−/−^ mice were fed a Western Diet for 14 weeks post-weaning (weaning at 3 weeks of age), before aortas were isolated and the plaque area was determined by ORO staining. **A:** Representative en face images of ORO staining of the inner curvature of the aortic arches. **B:** Quantification of ORO-stained area on inner curvature of the aortic arches from SGEF^+/+^ApoE^−/−^ and SGEF^−/−^ApoE^−/−^ (n = 10 per genotype; SGEF^+/+^ApoE^−/−^: 15.8±3.7%; SGEF^−/−^ApoE^−/−^: 8.8±3.7%; Data are mean ± SD. ***p<0.006. Bar graph at the right represents averaged measurements of all studied aortic arches. Left graph shows the measured ORO-stained area of individual aortas (represented by diamonds). **C and D:** As described under A and B, but for the descending thoracic aorta: **D:** Quantification of plaque area in the: SGEF^+/+^ApoE^−/−^: 3.2±1.9%; SGEF^−/−^ApoE^−/−^: 1.1±0.5%; Data are mean ± SD. ***p<0.007. **E:** Histology samples were stained for CD68 or ICAM-1 as indicated to detect macrophage presence in plaque areas. Graph at the right shows the quantification of CD68 positive areas in the plaque. n = 4. Asterisks indicate *p<0.05 according to Student T-test.

**Figure 5:**
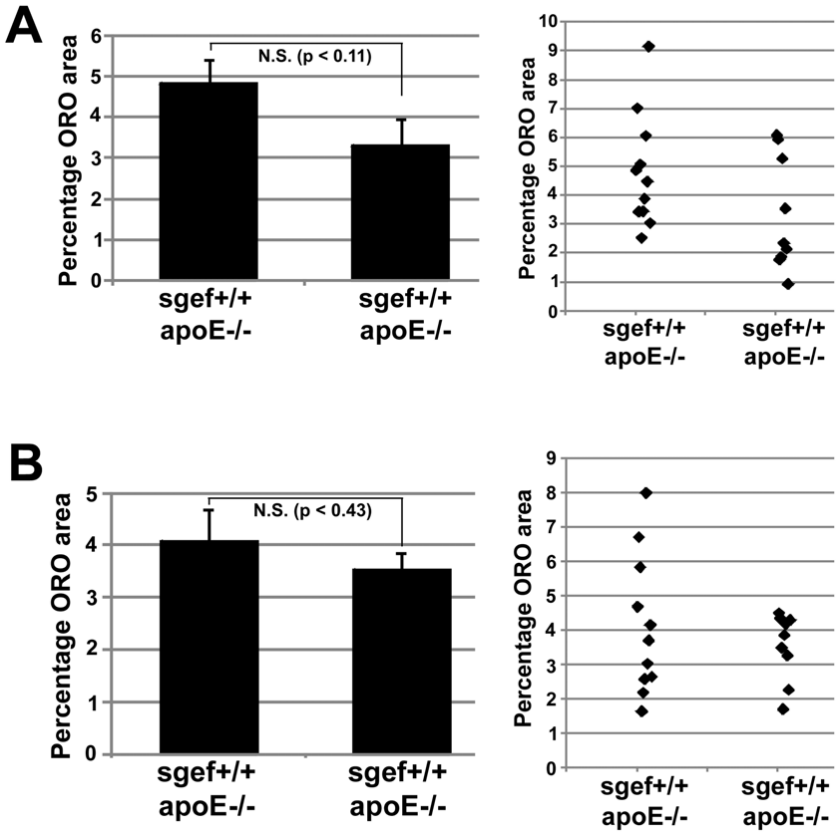
Atherosclerotic plaque formation in mice fed a normal chow diet. **A and B:** SGEF^+/+^ApoE^−/−^ and SGEF^−/−^ApoE^−/−^ mice were fed a chow diet for 16 weeks post weaning (weaning at 3 weeks of age), before aortas were isolated and the plaque area was determined by ORO staining (n = 11 for SGEF^+/+^ApoE^−/−^ aortas; n = 9 for SGEF^−/−^ApoE^−/−^ aortas; Data are mean ±SEM; N.S.: not significant; p-values represent 2-tailed Student's T-test with unequal variance). **A:** Quantification of ORO-stained area of the inner curvature of the aortic arch. The bar graphs at the left represent averaged measurements of all aortas. The right graph shows the measured ORO-stained area of individual aortas (represented by diamonds). SGEF^+/+^ApoE^−/−^: 4.8±2.0%; SGEF^−/−^ApoE^−/−^: 3.3±2.0%; p<0.11. **B:** Same as described under A for thoracic descending aorta: **B:** Descending thoracic aorta: SGEF^+/+^ApoE^−/−^: 0.8±0.4%; SGEF^−/−^ApoE^−/−^: 0.7±0.2%; p<0.43.

Leukocyte recruitment and transmigration through the endothelial cell layer into the vessel wall is a key step for the formation of atherosclerotic lesions [11]–[13]. Because ICAM-1 deficiency was previously found to protect from atherosclerosis in mice [2]–[4] and because SGEF acts downstream of ICAM-1 to facilitate the formation of docking-structures on endothelial cells [5], we sought to investigate the role of SGEF in a mouse atherosclerosis model. We generated experimental groups of SGEF^+/+^ApoE^−/−^ and SGEF^−/−^ApoE^−/−^ mice (Figure 3A). SGEF^+/+^ApoE^−/−^ and SGEF^−/−^ApoE^−/−^ did not exhibit differences in total body mass or blood pressure (Figure 3B and Table S2). Upon feeding a Western Diet (i.e. high fat and high cholesterol) for 14 weeks post-weaning, the control SGEF^+/+^ApoE^−/−^ mice developed prominent atherosclerotic plaques in their aortas, as measured by Oil Red O (ORO) staining. Under these same conditions, the areas of the ORO stained plaques were significantly smaller in SGEF^−/−^ApoE^−/−^ compared to SGEF^+/+^ApoE^−/−^ mice (Figure 4A–D). The decrease was observed in both the inner curvature of the aortic arch (area of ORO staining in SGEF^−/−^ApoE^−/−^ was decreased 45% of the area in the SGEF^+/+^ApoE^−/−^ aortas) and the descending thoracic aorta (area of ORO staining in SGEF^−/−^ApoE^−/−^ was decreased 66% of the area in the SGEF^+/+^ApoE^−/−^ aortas). Furthermore, immunohistochemical analysis showed that infiltrating CD68-positive macrophages were reduced in plaques of SGEF^−/−^ApoE^−/−^ mice (Figure 4E). Importantly, total body mass, triglyceride and cholesterol levels did not differ between the two experimental groups (Figure S2A–C). In parallel experiments, we measured the percentage of ORO stained areas when the atherosclerotic stimulus was reduced by feeding a regular chow diet instead of a Western Diet. Under these conditions, we observed a slight reduction of stained plaque areas in SGEF^−/−^ApoE^−/−^ compared to SGEF^+/+^ApoE^−/−^. For the inner curvature of the aortic arch, the plaque area in SGEF^−/−^ApoE^−/−^ was reduced by 31% compared to the plaque area in SGEF^+/+^ApoE^−/−^ aortas. In the thoracic descending aorta, the plaque area in SGEF^−/−^ApoE^−/−^ was reduced by 14% compared to the SGEF^+/+^ApoE^−/−^ aortas (Figure 5A–B). However, the reduction under this dietary condition was not statistically significant. Therefore, these data indicate that SGEF deficiency had most impact on the development of atherosclerosis when animals were exposed to a diet containing high cholesterol and high fat. It is noteworthy that studies on the influence of ICAM-1 deficiency during atherosclerosis in mice similarly found that certain experimental conditions (specifics of the diet, mouse background strain, age of study animals) did not result in significant plaque reductions [2]; [13].

**Figure 6:**
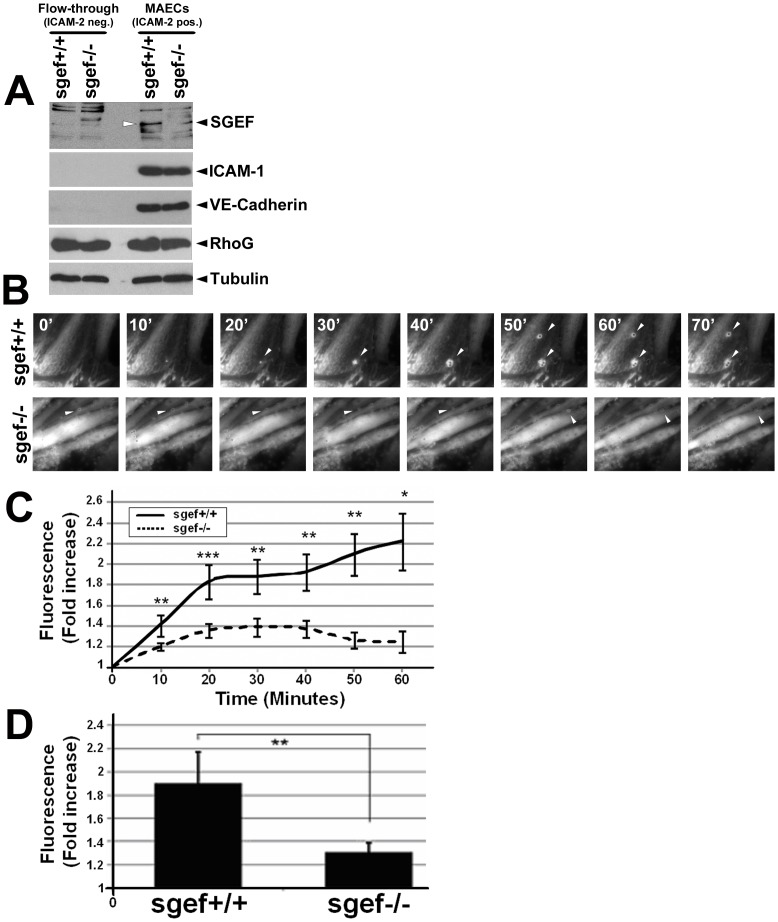
Defective activation of RhoG after ICAM-1 clustering on SGEF-deficient primary mouse aortic endothelial cells. **A:** Molecular characterization of the ICAM-2-positive cell isolation. All cells (flow-through fraction (ICAM-2 negative) and ICAM-2 positive) were stimulated with 10 ng/ml TNF-α for 4 hours prior to lysis for Western blotting as indicated. The ICAM-2-positive cells expressed ICAM-1 and VE-cadherin, markers for endothelial cells. Also, SGEF expression was only measured in the isolates from wildtype animals and not from the knock-out. Hereafter, the ICAM-2-positive cells are referred to as mouse aortic endothelial cells (MAECs). **B:** Representative time-lapse imaging of ELMO-YFP recruitment to sites of adhesion of anti-ICAM-1-Ab coated beads on TNF-α-treated MAECs from SGEF^+/+^ or SGEF^−/−^ animals. Arrowheads show bead location and only in the wildtype cells, ELMO recruitment is observed. Bar: 10 µm. **C** and **D:** Quantification of ELMO-YFP recruitment to adhered beads. Fluorescence intensity was measured in regions of adherent beads on MAECs, isolated from SGEF^+/+^ and SGEF^−/−^ animals. Graph **C** shows measurements at each time point. Data represent pooled results from 3 independent experiments. Graph **D** shows averaged fluorescent intensity at time-points from 10 to 60 minutes after bead binding. Data represent pooled results from 3 independent experiments. Asterisks indicate ***p<0.05, **p<0.005 or *p<0.0005 according to Student T-test.

To study if SGEF expression in hematopoietic cells, we isolated these cells from wildtype animals and permeabilized them to measure SGEF expression intracellularly using flow cytometry, since the limited number of cells did not allow us to quantify SGEF expression with Western blotting. We used isotype control antibodies as well as the SGEF-deficient leukocytes as threshold for the SGEF antibody staining. The data showed that SGEF was not expressed in murine hematopoietic cells, indicating that other cells were responsible for the reduced plaque formation in SGEF-deficient animals (Figure S3). Our previous work showed that RhoG is a major target of SGEF and mediates the formation of dorsal ruffles [10]. Additionally, our group showed that RhoG activation and formation of endothelial docking structures after ICAM-1 clustering was reduced when SGEF was silenced [5]. Therefore, we determined if RhoG activation upon ICAM-1 clustering was perturbed in SGEF-deficient murine endothelial cells. Primary cells from the intima of mouse aortas from SGEF^+/+^ and SGEF^−/−^ animals were isolated and subjected to magnetic cell-sorting for ICAM-2 expression. When cultured on fibronectin-coated surfaces, these cells (hereafter referred to as mouse aortic endothelial cells, MAECs) formed monolayers of elongated cells (Figure S4). Western blotting revealed that TNF-α-stimulated MAECs expressed ICAM-1 and VE-Cadherin, whereas the ICAM-2-negative cell population did not (Figure 6A), confirming the endothelial identity of the isolated MAECs. Due to the limited number of endothelial cells that can be isolated from mouse aortas, biochemical pull down assays using GST-ELMO to measure RhoG.GTP levels were not feasible. Therefore, through adenoviral delivery, YFP-tagged ELMO fragment was expressed in MAECs. Since ELMO is a specific downstream effector of RhoG, ELMO-YFP recruitment was measured as a read-out of local RhoG activation, similar to what has been described for local Rac1 activity upon phagocytosis [14]. TNF-α-stimulated primary MAECs from wildtype and SGEF-deficient animals were overlaid with polystyrene beads, coated with anti-ICAM-1 antibodies (hereafter: anti-ICAM-1 beads). When anti-ICAM-1 beads attached to the endothelium, time-lapse image acquisition was started. A representative example of ELMO-YFP recruitment to attached beads is shown in Figure 6B and supplemental movies (Movie S1 and S2). Quantification revealed reduced recruitment of ELMO-YFP to regions of attached beads in SGEF^−/−^ MAECs compared to SGEF^+/+^ cells (Figure 6C). Significant reduction in fluorescence intensity was measured in MAECs derived from SGEF^−/−^ compared to MAECs from SGEF^+/+^ mice at all time points between 0 and 60 minutes after bead attachment (Figure 6D).

**Figure 7:**
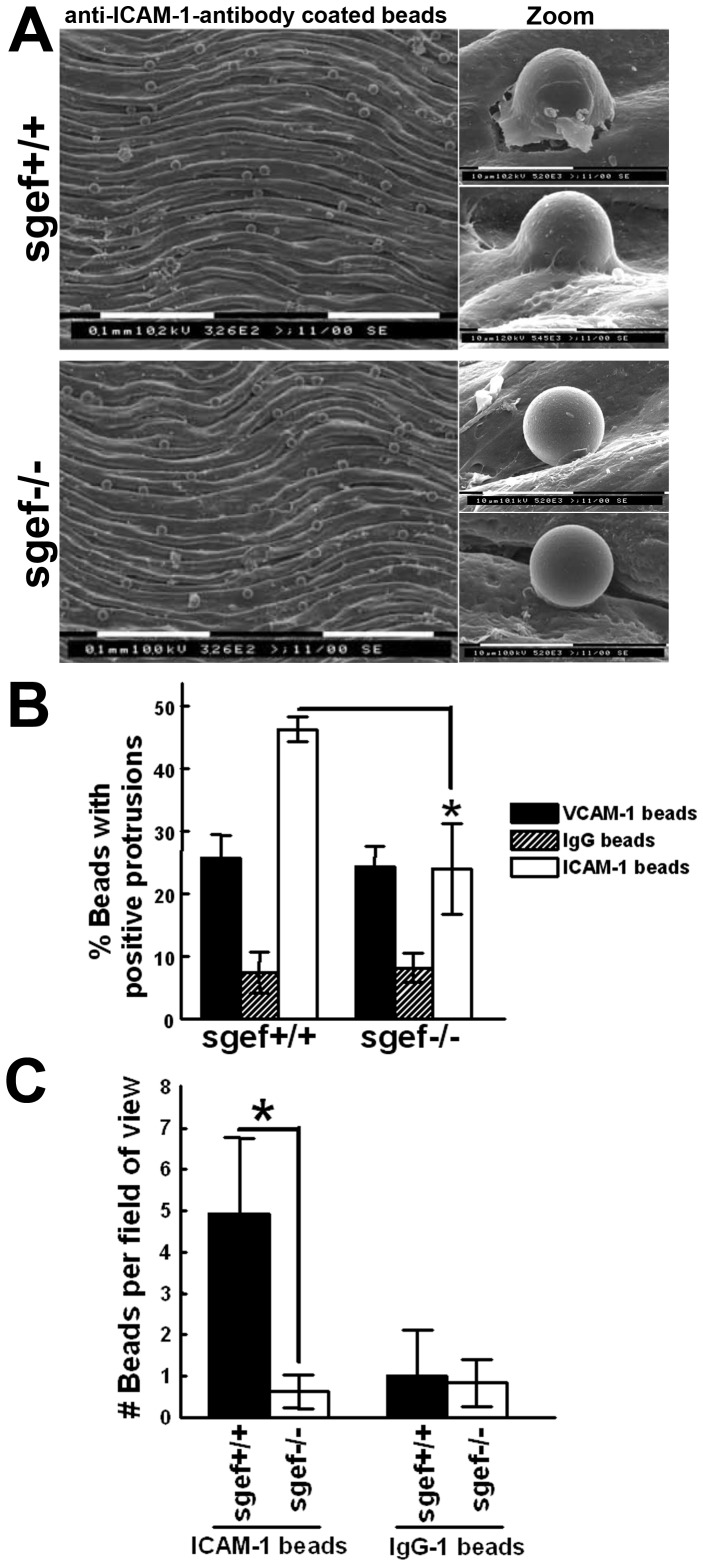
Membrane protrusions of intimal endothelial cells after ICAM-1 crosslinking. **A:** Isolated aortas from SGEF^+/+^ and SGEF^−/−^ mice were mounted with the intimal site facing up. After TNF-α treatment, the aortas were overlaid with anti-ICAM-1-Ab coated beads for 2 hours, before unbound beads were washed off and imaged by scanning electron microscopy. Representative scanning electron microscopy show attached anti-ICAM-1-Ab coated beads to the intimal surface of aortas from SGEF^+/+^ and SGEF^−/−^ animals. Right panels show magnification of adherent bead. Scale bars represent 0.1 mm or 10 µm, as indicated. **B:** Quantification of antibody coated beads that induced membrane protrusions on aortas from SGEF^+/+^ and SGEF^−/−^ animals. Anti-ICAM-1-Ab coated beads: SGEF^+/+^: 53.3±14.8%; SGEF^−/−^: 37.3±14.8%. Anti-VCAM-1-Ab coated beads: SGEF^+/+^: 25.8±8.9%; SGEF^−/−^: 24.2±10.0%. Anti-IgG1-Ab coated beads: SGEF^+/+^: 8.2±7.0%; SGEF^−/−^: 7.5±9.3%. Data are mean ±SEM. ***p<0.001. C: Quantification of total number of anti-ICAM-1-Ab or IgG1-Ab coated beads that adhered to the intimal surfaces of isolated and intact carotid arteries under flow conditions (5 dynes/cm^2^). Anti-ICAM-1-Ab coated beads: SGEF^+/+^: 4.9±1.8; SGEF^−/−^: 0.6±0.4. Anti-IgG1-Ab coated beads: SGEF^+/+^: 1.0±1.1; SGEF^−/−^: 0.8±0.6. Experiment was carried out at least 4 times. Data are mean ±SEM. ***p<0.001.

Our previous work showed that silencing of SGEF specifically affected ICAM-1-mediated formation of docking structures around adhered leukocytes [5]. Therefore, we examined if SGEF-deficiency in mice decreased docking structure formation on endothelial cells *in situ*. Aortas were isolated from SGEF^+/+^ and SGEF^−/−^ mice as described in the Methods section and stimulated with TNF-α overnight. Next, anti-ICAM-1 beads were allowed to adhere to the endothelial surface of the aortas for 2 hours. Scanning electron microscopy (EM) analysis revealed that the aortic intima, i.e. the endothelium, from wildtype animals induced membrane protrusions around adherent beads, whereas the aortic endothelium from SGEF^−/−^ animals showed minimal protrusion activity in response to the beads (Figure 7A). Quantification demonstrated that the percentage of anti-ICAM-1 beads with membrane protrusions on their surface was significantly decreased on aortas from SGEF^−/−^ animals compared to SGEF^+/+^ aortas (SGEF^+/+^: 53.3±14.8%; SGEF^−/−^: 37.3±14.8%; Figure 7B). Importantly, no differences were observed between the genotypes when beads coated with anti-VCAM-1 or nonspecific IgG1 antibodies were used (Figure S5A and S5B). When the total number of anti-ICAM-1 beads that were attached to aortas (independent of membrane protrusions) was counted, no difference in bead number between aortas isolated from SGEF^+/+^ and SGEF^−/−^ animals was detected (Figure S5C).

These data indicated that adhesion to endothelial ICAM-1 under static conditions was not affected by SGEF-deficiency. However, it has been proposed that docking structures provide mechanical support for leukocytes to resist flow forces during adhesion [15]; [16]. Therefore, anti-ICAM-1 beads were injected into the lumen of isolated intact carotid arteries that were attached to a perfusion system, as described in the Materials and Methods section. Next, the arteries were continuously perfused with 5 dynes/cm^2^ for 5 minutes. Data from the literature shows that the shear rates in arteries are usually 10 dynes/cm^2^ or more [17]; [18]. However, due to technical difficulties, we were unable to increase the shear rate in the isolated arteries above 5 dynes/cm^2^. Quantification analysis revealed that significantly lower number of beads adhered to the inner surface of flow-exposed carotid arteries from SGEF^−/−^ mice compared to SGEF^+/+^ mice (SGEF^+/+^: 4.92 SD±1.85, SGEF^−/−^: 0.63 SD±0.41; Figure 7C). Beads coated with an IgG1-isotype control antibody adhered very poorly under flow conditions (Figure 7C). We wish to acknowledge that our set-up did not allow the perfusion of isolated immune cells over the arteries, due to firm adhesion of leukocytes to the inner surface of the cannula, resulting in obstruction. Taken together, these results showed that the formation of functional docking structures upon ICAM-1 clustering is strongly reduced in SGEF-deficient arteries.

## Discussion

In this study, we have implicated a Rho GEF, SGEF, in the formation of atherosclerosis *in vivo*. Our data show a significant reduction in atherosclerotic lesions in the aortas of SGEF-deficient mice when fed a Western diet. At the molecular level, we show that SGEF-deficiency decreased RhoG activation and the formation of docking structures in response to ICAM-1 clustering. Rahaman and colleagues have shown that the Rho-GEF Vav1 is involved in the formation of foam cells and thereby contributes to the development of atherosclerosis [19]. To our knowledge, this is the first description of an endothelial Rho GEF involved in the formation of atherosclerosis. SGEF was targeted by deleting exon 4 and 5. Overexpressing this truncated form of the protein showed no functionality. Moreover, using SGEF specific antibodies that recognize the truncated form, we found that the mutant protein was not translated in mice. Therefore, we concluded that the SGEF deletion strategy resulted in mice that did not show any off-target phenotypes.

Atherosclerosis is nowadays recognized as an inflammatory disease [20]. Inflammation is characterized by activated leukocytes that extravasate from the vessel into the underlying tissue. During the development of atherosclerosis, this process occurs inappropriately [21]. Monocytes are the first immune cells that are detected in early atherosclerotic lesions [1]. In this study, mice deficient for SGEF in all cell types are generated. Therefore, the reduced plaque areas that were detected in SGEF-deficient mice could potentially also be the result of less infiltrated leukocytes, in particular monocytes, due to the lack of SGEF in these cells. SGEF is expressed in epithelial and endothelial cells [5]; [10] but we have not detected in mouse monocytes or neutrophils. In previous work, no expression was detected in HL60 and U937 cells [10]. These cells are hematopoietic precursor cells for neutrophils and monocytes, respectively. These data suggest that primary monocytes and neutrophils are devoid of SGEF expression and indicate that the decrease in atherosclerotic plaques in the SGEF-deficient animals is due to the absence of SGEF in endothelial cells rather than in monocytes.

Recent studies have shown that the endothelium actively participates in the extravasation process by inducing docking structures [5]–[7]; [21]; [22]. These are characterized by membrane protrusions, also known as dorsal membrane ruffles that extend from the apical surface of the endothelium. The small GTPases Rac1 and in particular RhoG are recognized for their ability to induce these type of membrane extension [10]. Also during leukocyte binding to the endothelium, Rac1 and RhoG are activated [23]. Our work indicated that reduction of RhoG expression represses the ability of the endothelium to induce dorsal ruffles and, as a consequence, also the formation of docking structures around adherent leukocytes [23]. Additional experiments indicate that the RhoG-specific Rho-GEF SGEF is responsible for the GTP exchange on RhoG in these endothelial cells during leukocyte adhesion and transmigration [5]. Our data presented here show that endothelial cells from aortas that lack SGEF compared with wildtype endothelial cells fail to induce these typical docking structures. Notably, we found that the SGEF−/− aortas were less able to retain beads coated with anti-ICAM-1 under conditions of flow. This is consistent with the docking structures normally functioning to prevent leukocytes from being washed away from regions of inflammation. Together, these data indicate that the reduced lesion area in SGEF-deficient animals is most likely due to the inability of the endothelium to induce docking structures that actively retain leukocytes against the forces of blood flow thereby assisting their crossing of the vessel wall.

Our data suggest that SGEF may be an attractive pharmacological target for decreasing atherosclerosis, since we have not observed any negative phenotypes in mice that lack SGEF. An SGEF-based pharmacological prevention strategy is further supported by studies showing that ICAM-1 deficiency also results in reduced atherosclerosis [2]–[4]. Therapies targeting ICAM-1 are an interesting approach; however, adverse effects were found when anti-ICAM-1 antibodies were used in a phase III clinical study with ischemic stroke patients [24]. In general, antibody-based therapies targeting cell surface receptors involve serious side effects [25] and are less appropriate for long term applications such as treatment of atherosclerosis. Additionally, ICAM-1 is involved in acute host defense, and therefore increased susceptibility to infectious organisms is an intrinsic side effect of targeting this receptor [26]. Developing therapeutic drugs targeting SGEF function may avoid these problems.

## Materials and Methods

### Mice

To build a targeting construct for the murine SGEF gene, the pOSfrt-loxP vector was used (kindly provided by Dr. Oliver Smithies and the UNC Animal Model Core Facility). Mouse genomic areas were amplified by PCR. The short arm: (3039 bp, starting 470 bp downstream of exon 5; ending 3509 bp downstream of exon 5) was cloned into the *Bam*HI/*Not*I sites of the vector. A double stranded oligo-nucleotide representing the second LoxP site and an *Fse*I site was introduced into the *Nhe*I/*Cla*I sites of the construct. The DNA fragment of the genomic area covering Exon 4/5 (4495, starting 624 bp upstream of exon 4 immediately after a genomic *Apa*I site; ending 467 bp downstream of exon 5) was cloned into the *Cla*I/*Fse*I sites of the construct. Finally, the long arm (5412 bp, starting 6042 bp upstream of exon 4; ending 630 bp upstream of exon 4 immediately before a genomic *Apa*I site) was cloned via the *Nhe*I site of the construct and its orientation was checked by sequencing. For ES cell transfection, the construct was linearized by a *Not*I digest. ES cell transfections and blastocyst injection were performed by the UNC Animal Model Core Facility. ES cell clones that have integrated the construct into the genome through homologous recombination of the short and long arm were identified by Southern blotting. Two selected clones were then transfected with an expression construct for cre-recombinase to delete exon 4/5, which was again confirmed by Southern blotting. These cells were used for blastocyst injection and the mutated allele was crossed out by breeding chimeras with wild type c57/black6 mice. Animals were crossed back to wild type c57/black6 mice over at least 7 generations. Wildtype c57/black6 mice (strain: C57BL/6J) and *ApoE*-deficient mice (strain: B6.129P2-*ApoE^tm1Unc^*/J) were obtained from Jackson Laboratories. Atherosclerosis study mice were either fed a regular chow diet (‘Prolab RMH3000’, LabDiet 6.4% fat, 195 ppm cholesterol) or a high fat diet (‘Western Diet for Rodents’, Testdiet, 19.9% fat, 1707 ppm cholesterol). Animals used in the atherosclerosis study were all males. For the atherosclerosis study, animals were backcrossed at least 7 times. For the isolated vessels, animals were backcrossed at least 10 times. All animal experiments described here are approved by the Institutional Animal Care and Use Committee of the University of North Carolina (IACUC-ID: 06-145, 08-202, 09-043) and the Local Ethical Committee of the University of Amsterdam (NIH Publication No. 85–23, revised 1996).

### Oil Red O (ORO) staining method

Mice were perfused with PBS followed by PBS/4% PFA. Dissected aortas were further fixed for 48 hours in PBS/4% PFA. After incubating the aortas in 75% ethanol the aortas were stained with 0.5% Oil Red O (ORO)/60% isopropanol. Images were taken of longitudinally opened aortas that were pinned down on wax. Images were taken with dissection microscope and a color camera. Percentage of ORO stained areas was determined with ImageJ software.

### Antibodies and Reagents

The following antibodies were used: mouse anti-RhoG (clone 1F3, Millipore); rat-anti-mouse-ICAM2 (clone 3C4, BD-Pharmingen); rat-anti-mouse-ICAM1 (clone MAB796, R&D); mouse-anti-VE-Cadherin (clone F8, Santa Cruz), anti-VCAM-1 mAb (R&D), IgG1-isotype control Ab (Sanquin, Amsterdam, the Netherlands), anti-Myc mAb (Clone 9E10; Sigma) and secondary HRP Abs were from Dako (Denmark). Phalloidin-Alexa488 or Texas-red was purchased from Invitrogen. Endothelial tissue culture media EBM-2 (supplemented with EGM-2 SingleQuots™) was obtained from Lonza. DMEM media was obtained from Gibco. The following reagents were used: Oil Red O (Sigma); Heparin (Abraxis, 10000 USP units/ml); magnetic beads coated with sheep-anti-rat-IgG (Dynal); polystyrene beads ‘Amino Microspheres’ (Polyscience 3.0 µm carotid artery perfusion, 5 μm for biosensor experiments, 10 µm for aorta EM experiments). Proteins were bound to polystyrene beads according to the manufacturer's protocol followed by free glutaraldehyde group blocking with 0.5 M ethanolamine and 2 mg/ml BSA.

### Isolation of Mouse Aortic Endothelial Cells

Mouse aortic endothelial cells (MAECs) were isolated by combining two modified protocols that have been described previously [27]; [28]. Briefly, adult mice (8 to 12 weeks old) were anesthetized and then perfused with PBS containing 20% FBS and 100 units heparin. The thoracic descending aorta was isolated (from end of descending arch to diaphragm) and put into a glass dish with the bottom covered with a layer of sterile wax. All subsequent dissecting steps were done in this dish under DMEM media. Remaining connecting tissue on the outside of the aorta was removed before the aorta was opened by a longitudinal cut. The opened aorta was pinned down with small needles with the intimal surface facing up. After removing the media, the pinned down aorta was overlaid with 0.5 ml of DMEM/Collagenase (2 mg/ml) at 37°C for 60 minutes. Subsequently, continuous flushing of the intima released the aortic endothelial cells which were then plated on collagen-I coated dishes. After two days, the cells were overlaid with magnetic Dynal beads that had been coated with anti-ICAM2 antibody. After 30 minutes the cells were trypsinized and sorted by a magnet, before the bead-bound cells were again plated on Collagen-1 coated dishes and used for experiments within the following 7 to 14 days.

### Biosensor to Measure Local RhoG activation

To calculate local activation of RhoG in cells, a fluorescently tagged fragment of ELMO1 was used (ELMO-Ypet). The cDNA was cloned into pAd vector and adenovirus particles were produced in 293A cells (‘ViralPower Adenoviral Expression System’ from Invitrogen). Twenty-four hours post infection of MAECs; time-lapsed image stacks were acquired. Fluorescence was measured in a circular area (diameter 8.3 µm) around an attached bead. Fluorescence right before bead attachment was used as the baseline fluorescence.

### Bead Adhesion Assay and Scanning Electron Microscopy of Mouse Aortas

Murine aortas were dissected using microsurgery, cut open longitudinally and kept overnight at 37°C at 5% CO_2_. In parallel, the samples were pretreated with 10 ng/mL murine TNF-alpha overnight. For scanning electron microscopy (scanning EM), 1 μg/ml of antibody-containing beads was washed and re-suspended in EGM-2 medium. Next, the beads were incubated on an isolated murine aorta for 2 hours as indicated. After the appropriate time, unbound beads were removed, and samples were put on ice, gently washed three times with ice-cold PBS containing 1 mM Ca^2+^/Mg^2+^. Subsequently samples were fixed in 2.5% glutaraldehyde/PBS for 30 min at room temperature, and processed for scanning electron microscopy as described previously ^6^. Cells were examined on a scanning electron microscope (model 820; JEOL) at 15 kV. Scanning EM analysis revealed the induction of docking structures as described in results section.

### Perfusion assay

Specially-developed cannulae are placed on each end of an isolated carotid artery and EGM-2 medium, containing anti-ICAM-1 or IgG1-isotype Ab-coated beads, was added. Beads were allowed to adhere for 30 minutes, followed by induction of flow at 5 dynes/cm^2^. After 5 minutes, arteries were fixed using 3.7% formaldehyde, permeabilized using 0.2% Triton X-100, stained for F-actin using Texas-Red Phalloidin and nuclei using DAPI and analyzed by confocal laser scanning microscopy. Beads were visualized using DIC. Tile scan (3×3) images were recorded with a ZEISS LSM510 confocal microscope with appropriate filter settings and the number of beads was counted per tile scan. At least 3 tile scans were counted per experiment.

### Statistical analysis

Statistical comparisons between experimental groups were performed by the 2-tailed Student's T-test with unequal variance. A 2-tailed p-value of <0.05 was considered significant.

## Supporting Information

Figure S1 **Observed genotype frequency of offspring originating after breeding SGEF^+/−^ mice (134 pups from 20 litters, backcross generation ≥7).**
(TIF)

Figure S2 **Characterization of plasma samples from study animals at the final age of animals 14** **weeks post-weaning.** **A:** Total cholesterol levels in mg/dL in plasma. n = 5 per genotype. Data are mean ±SEM. **B:** Triglyceride levels in mg/dL in plasma. n = 5 per genotype. Data are mean ±SEM. **C:** Total body mass (grams) of male mice fed a Western Diet over 14 weeks. Closed line represents SGEF^+/+^ApoE^−/−^ animals; Dotted line represents SGEF^−/−^ApoE^−/−^ animals. n = 5 per genotype.
(TIF)

Figure S3 **SGEF expression analysis in hematopoietic cells.** Hematopoietic cells were isolated from whole blood from wildtype and SGEF-deficient mice and subsequently fixed and permeabilized for intracellular staining. Cells were incubated subsequently with SGEF polyclonal Ab (Proteintech Europe, Manchester, UK) and secondary FITC-labeled Ab. The Ab epitope of human SGEF, to which the Ab was directed, shows 98% homology with murine SGEF. Forward-side scatter plot shows distribution of all cells. P1 region reflects immune cells and show very few positive cells in both wildtype and SGEF-deficient samples. Purple area reflects platelets, which were also negative for SGEF staining in both conditions. Graph below shows the quantification of mean fluorescent intensity (MFI) of intracellular SGEF staining. Four animals were analyzed. Data are mean ± SEM.
(TIF)

Figure S4 **Phase microscopy images of isolated endothelial monolayers.** ICAM-2-positive cells were isolated from the intimal side of the mouse aortas from *SGEF* ^+/+^ and *SGEF* ^−/−^ mice. The dark particles are ICAM-2-coated magnetic Dynal beads that remained after cell sorting. Scale bar: 10 µm.
(TIF)

Figure S5 **Membrane protrusions of intimal endothelial cells after VCAM-1 clustering or control beads.** **A:** Isolated aortas from SGEF^+/+^ and SGEF^−/−^ mice were mounted with the intimal site facing up. After TNF-α treatment, the aortas were overlaid with anti-VCAM-1-Ab coated beads for 2 hours, before unbound beads were washed off and imaged by scanning electron microscopy. Representative scanning electron microscopy show attached anti-VCAM-1-Ab coated beads to the intimal surface of aortas from SGEF^+/+^ and SGEF^−/−^ animals. Right panels show magnification of adherent bead. Scale bars represent 0.1 mm or 10 µm, as indicated. **B:** Same as described under A, but VCAM-1 beads were replaced by IgG1-isotype Ab coated beads. **C:** Adhesion of anti-ICAM-1-Ab coated beads to the intimal surface of aortas from SGEF^+/+^ and SGEF^−/−^ animals was similar under static conditions. SGEF^+/+^: 16.7±6.9 beads per field, SGEF^−/−^: 15.4±6.8 beads per field. Data are mean ±SEM.
(TIF)

Table S1 **Analysis of SGEF^−/−^ animals.** Differential leukocyte counts of male and female SGEF^+/+^ and SGEF^−/−^ mice (backcross generation ≥7).
(TIF)

Table S2 **Intra aortic blood pressure measurements.** Animals were measured 19 weeks of age. ± represents SD; N.S.: not significant.
(TIF)

Movie S1 Movie of ELMO-YFP recruitment to sites of attached anti-ICAM-1 beads on TNF-α-treated MAECs isolated from SGEF^+/+^ animals. The arrow indicates the location on an attaching anti-ICAM-1 bead.
(AVI)

Movie S2 Movie of ELMO-YFP recruitment to sites of attached anti-ICAM-1 beads on TNF-α-treated MAECs isolated from SGEF^−/−^ animals. The arrow indicates the location on an attaching anti-ICAM-1 bead.
(AVI)
