# Supplementary figures and images for: The Guanine-Nucleotide Exchange Factor SGEF Plays a Crucial Role in the Formation of Atherosclerosis
Source: PLoS One. 2013 Jan 25;8(1):e55202. doi: 10.1371/journal.pone.0055202 (PMC3555862; doi:10.1371/journal.pone.0055202)

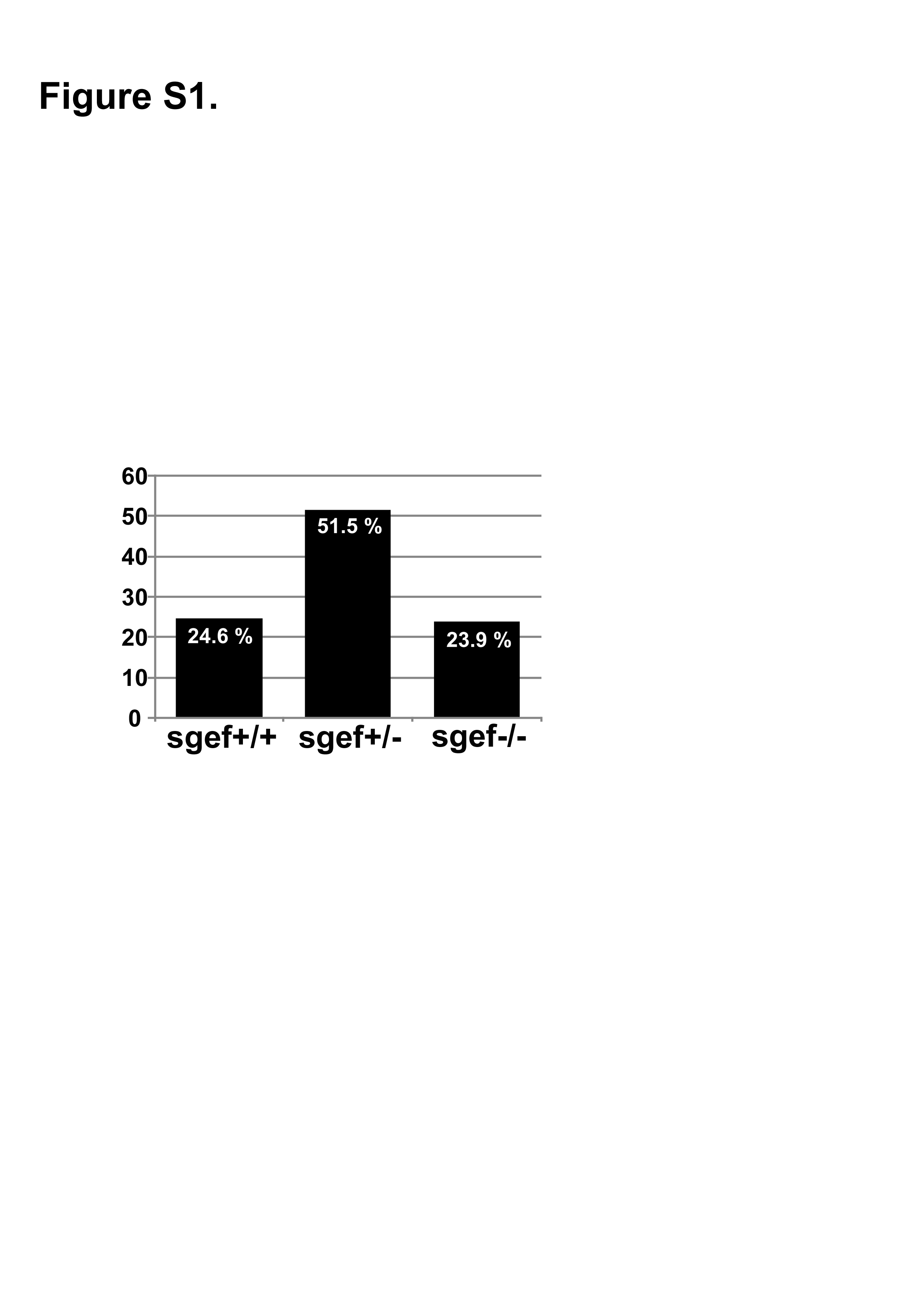

Supplement: Figure S1 — Observed genotype frequency of offspring originating after breeding SGEF+/− mice (134 pups from 20 litters, backcross generation ≥7). (TIF) [file pone.0055202.s001.tif]

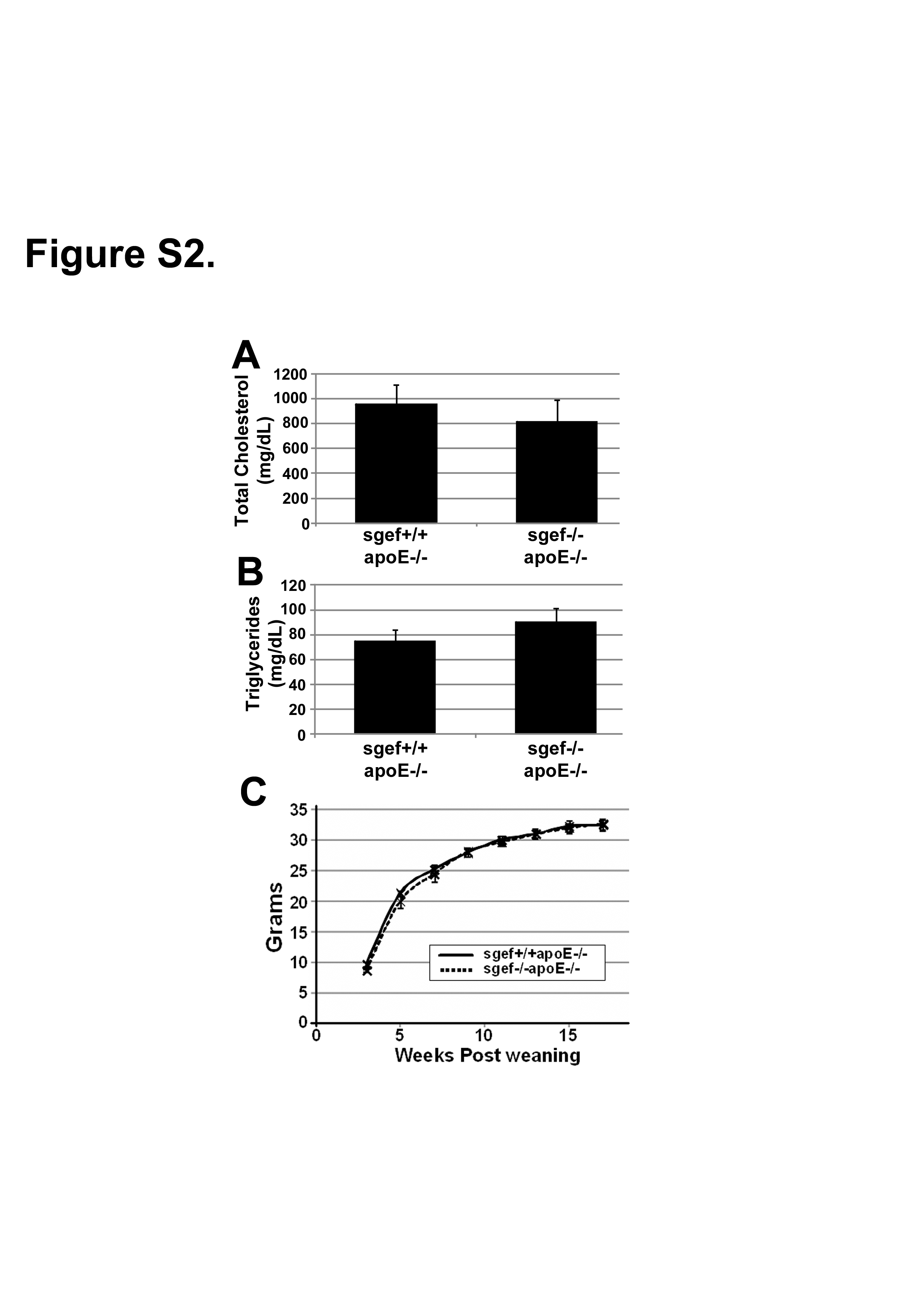

Supplement: Figure S2 — Characterization of plasma samples from study animals at the final age of animals 14 weeks post-weaning. A: Total cholesterol levels in mg/dL in plasma. n = 5 per genotype. Data are mean ±SEM. B: Triglyceride levels in mg/dL in plasma. n = 5 per genotype. Data are mean ±SEM. C: Total body mass (grams) of male mice fed a Western Diet over 14 weeks. Closed line represents SGEF+/+ApoE−/− animals; Dotted line represents SGEF−/−ApoE−/− animals. n = 5 per genotype. (TIF) [file pone.0055202.s002.tif]

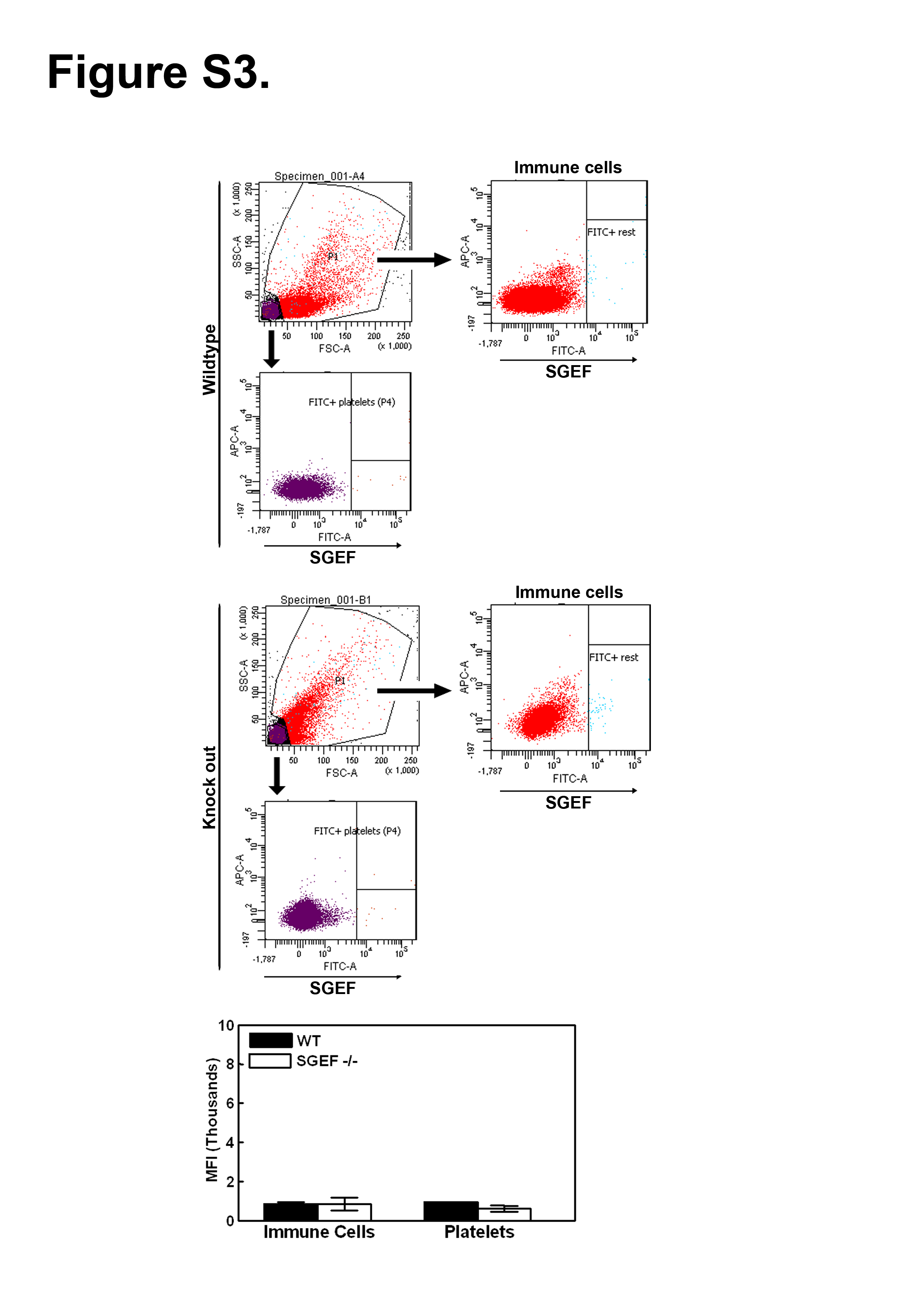

Supplement: Figure S3 — SGEF expression analysis in hematopoietic cells. Hematopoietic cells were isolated from whole blood from wildtype and SGEF-deficient mice and subsequently fixed and permeabilized for intracellular staining. Cells were incubated subsequently with SGEF polyclonal Ab (Proteintech Europe, Manchester, UK) and secondary FITC-labeled Ab. The Ab epitope of human SGEF, to which the Ab was directed, shows 98% homology with murine SGEF. Forward-side scatter plot shows distribution of all cells. P1 region reflects immune cells and show very few positive cells in both wildtype and SGEF-deficient samples. Purple area reflects platelets, which were also negative for SGEF staining in both conditions. Graph below shows the quantification of mean fluorescent intensity (MFI) of intracellular SGEF staining. Four animals were analyzed. Data are mean ± SEM. (TIF) [file pone.0055202.s003.tif]

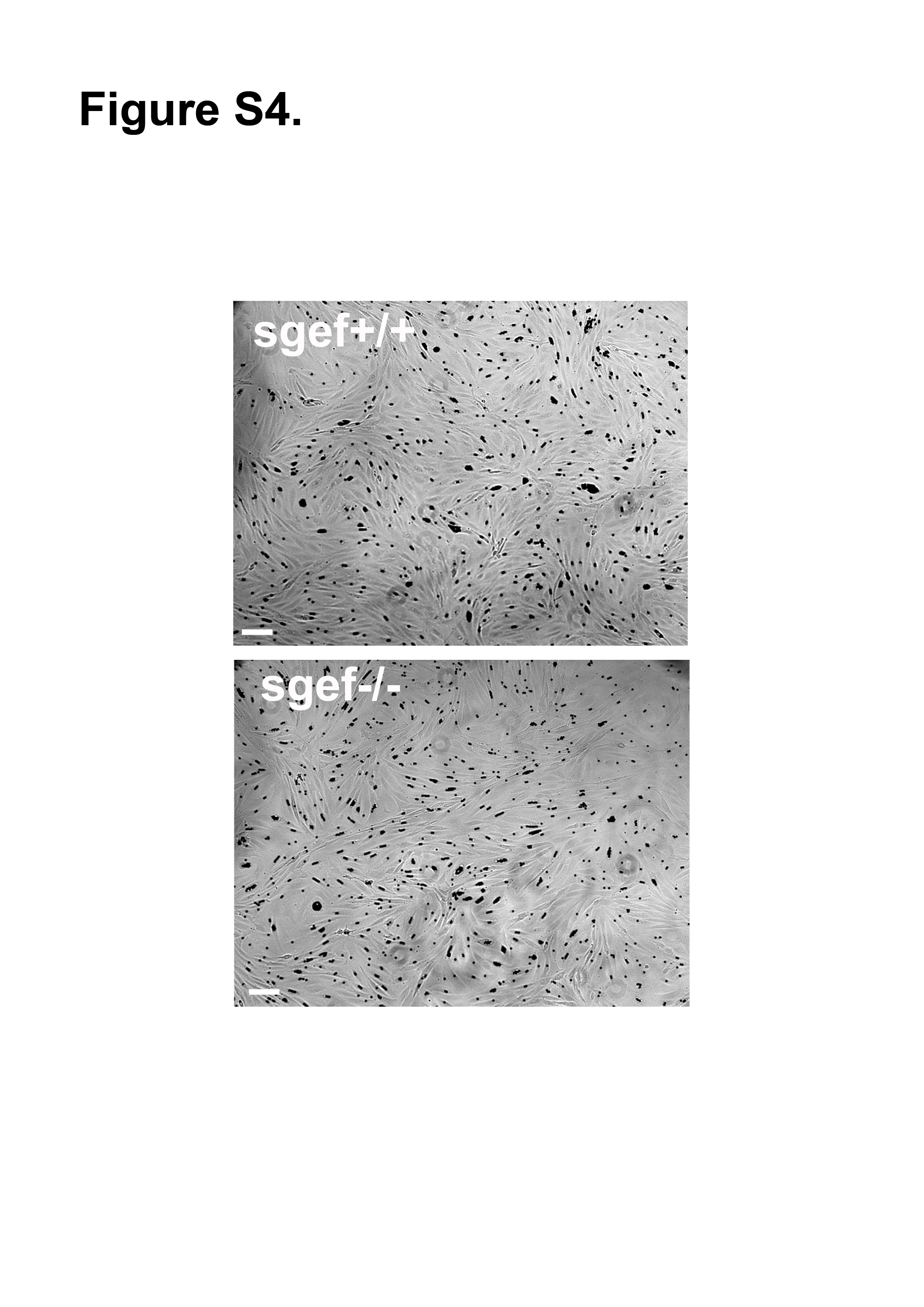

Supplement: Figure S4 — Phase microscopy images of isolated endothelial monolayers. ICAM-2-positive cells were isolated from the intimal side of the mouse aortas from SGEF +/+ and SGEF −/− mice. The dark particles are ICAM-2-coated magnetic Dynal beads that remained after cell sorting. Scale bar: 10 µm. (TIF) [file pone.0055202.s004.tif]

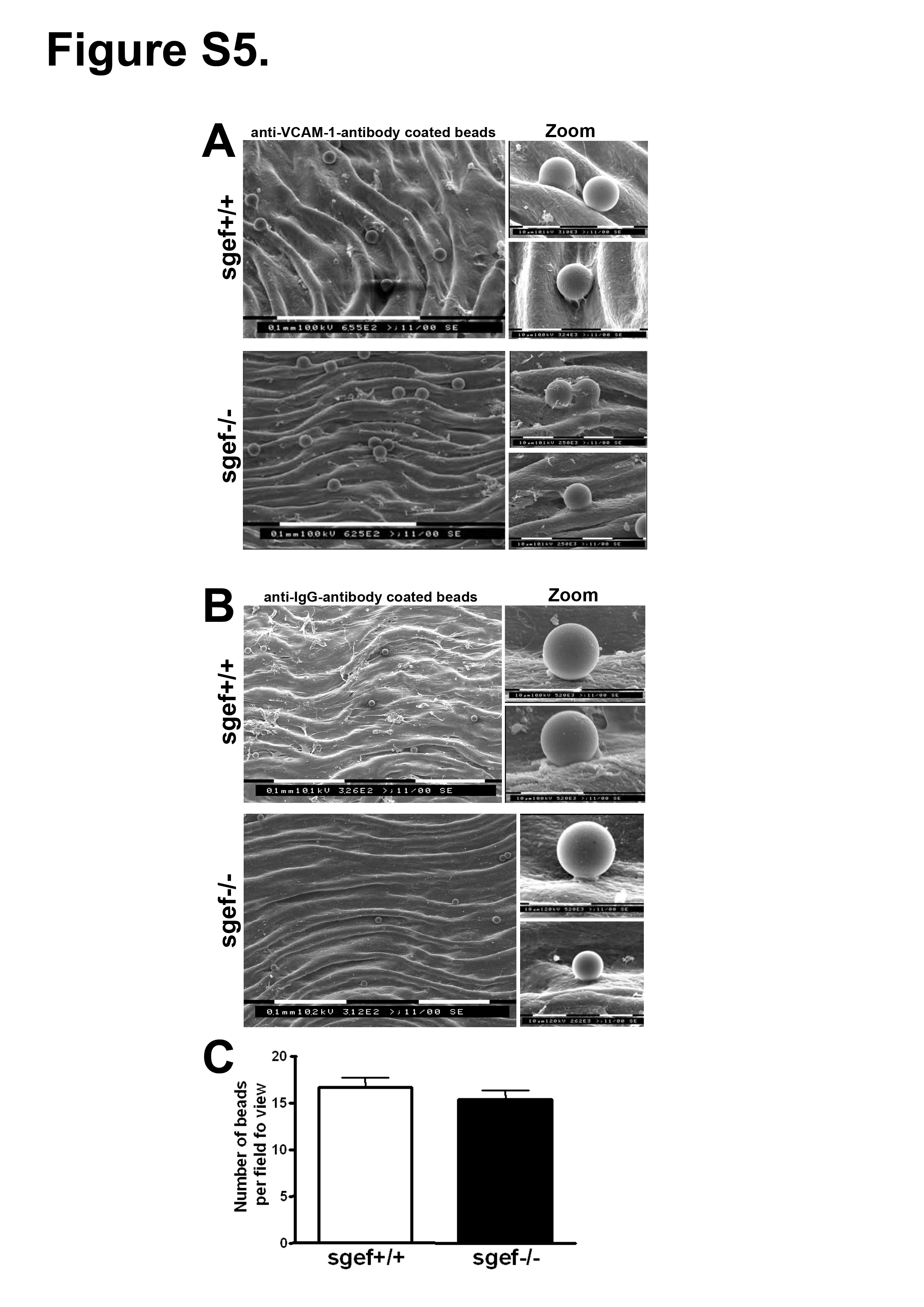

Supplement: Figure S5 — Membrane protrusions of intimal endothelial cells after VCAM-1 clustering or control beads. A: Isolated aortas from SGEF+/+ and SGEF−/− mice were mounted with the intimal site facing up. After TNF-α treatment, the aortas were overlaid with anti-VCAM-1-Ab coated beads for 2 hours, before unbound beads were washed off and imaged by scanning electron microscopy. Representative scanning electron microscopy show attached anti-VCAM-1-Ab coated beads to the intimal surface of aortas from SGEF+/+ and SGEF−/− animals. Right panels show magnification of adherent bead. Scale bars represent 0.1 mm or 10 µm, as indicated. B: Same as described under A, but VCAM-1 beads were replaced by IgG1-isotype Ab coated beads. C: Adhesion of anti-ICAM-1-Ab coated beads to the intimal surface of aortas from SGEF+/+ and SGEF−/− animals was similar under static conditions. SGEF+/+: 16.7±6.9 beads per field, SGEF−/−: 15.4±6.8 beads per field. Data are mean ±SEM. (TIF) [file pone.0055202.s005.tif]

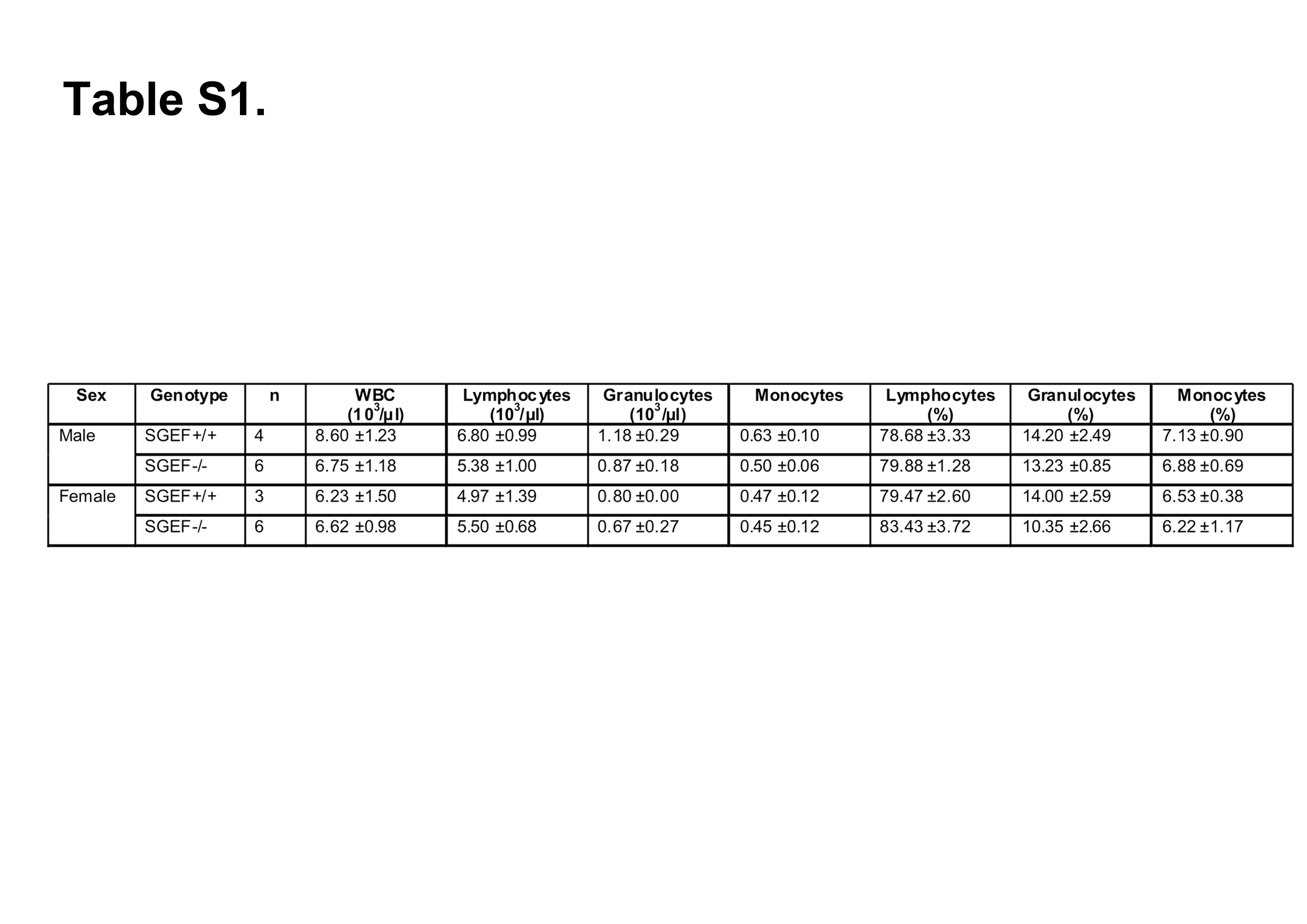

Supplement: Table S1 — Analysis of SGEF−/− animals. Differential leukocyte counts of male and female SGEF+/+ and SGEF−/− mice (backcross generation ≥7). (TIF) [file pone.0055202.s006.tif]

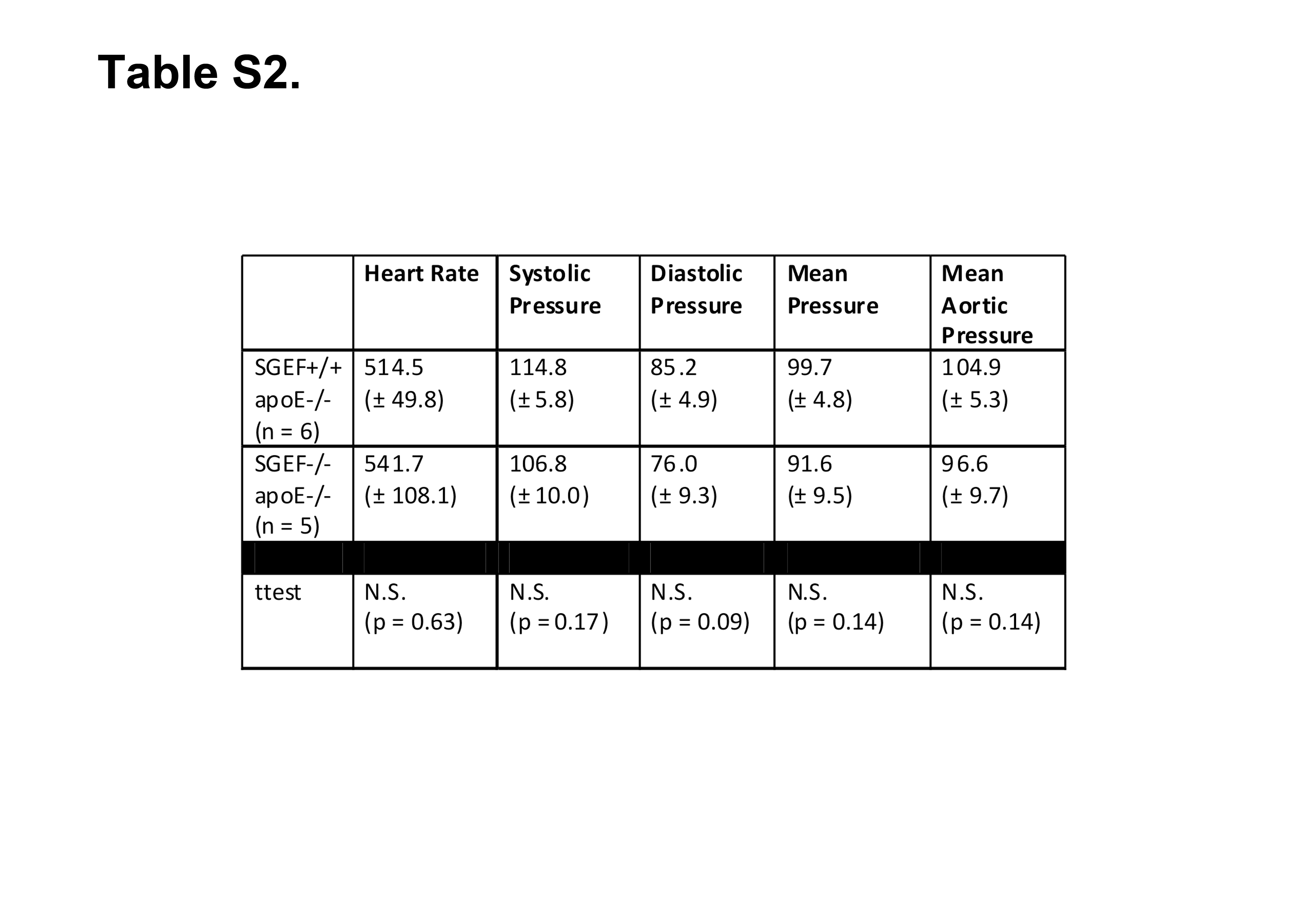

Supplement: Table S2 — Intra aortic blood pressure measurements. Animals were measured 19 weeks of age. ± represents SD; N.S.: not significant. (TIF) [file pone.0055202.s007.tif]
